# Circulating Vitamin D Concentrations and Risk of Atrial Fibrillation: A Mendelian Randomization Study Using Non-deficient Range Summary Statistics

**DOI:** 10.3389/fnut.2022.842392

**Published:** 2022-06-17

**Authors:** Nan Zhang, Yueying Wang, Ziliang Chen, Daiqi Liu, Gary Tse, Panagiotis Korantzopoulos, Konstantinos P. Letsas, Christos A. Goudis, Gregory Y. H. Lip, Guangping Li, Zhiwei Zhang, Tong Liu

**Affiliations:** ^1^Tianjin Key Laboratory of Ionic-Molecular Function of Cardiovascular Disease, Department of Cardiology, Tianjin Institute of Cardiology, Second Hospital of Tianjin Medical University, Tianjin, China; ^2^Kent and Medway Medical School, Canterbury, United Kingdom; ^3^Epidemiology Research Unit, Cardiovascular Analytics Group, Hong Kong, China; ^4^First Department of Cardiology, University of Ioannina Medical School, Ioannina, Greece; ^5^Arrhythmia Unit, Laboratory of Cardiac Pacing and Electrophysiology, Onassis Cardiac Surgery Center, Athens, Greece; ^6^Department of Cardiology, Serres General Hospital, Serres, Greece; ^7^Liverpool Centre for Cardiovascular Science, University of Liverpool and Liverpool Heart and Chest Hospital, Liverpool, United Kingdom; ^8^Department of Clinical Medicine, Aalborg University, Aalborg, Denmark

**Keywords:** vitamin D, Mendelian randomization, single-nucleotide polymorphisms, causal association, atrial fibrillation

## Abstract

**Background and Aims:**

Vitamin D deficiency is a common disorder and has been linked with atrial fibrillation (AF) in several observational studies, although the causal relationships remain unclear. We conducted a Mendelian randomization (MR) analysis to determine the causal association between serum 25-hydroxyvitamin D [25(OH)D] concentrations and AF.

**Methods and Results:**

The analyses were performed using summary statistics obtained for single-nucleotide polymorphisms (SNPs) identified from large genome-wide association meta-analyses conducted on serum 25(OH)D (*N* = 79,366) and AF (*N* = 1,030,836). Six SNPs related to serum 25(OH)D were used as instrumental variables. The association between 25(OH)D and AF was estimated using both the fixed-effect and random-effects inverse variance weighted (IVW) method. The MR analyses found no evidence to support a causal association between circulating 25(OH)D level and risk of AF using random-effects IVW (odds ratio per unit increase in log 25(OH)D = 1.003, 95% CI, 0.841–1.196; *P* = 0.976) or fixed-effect IVW method (OR = 1.003, 95% CI, 0.876–1.148; *P* = 0.968). Sensitivity analyses yielded similar results. No heterogeneity and directional pleiotropy were detected.

**Conclusion:**

Using summary statistics, this MR study suggests that genetically predicted circulating vitamin D concentrations, especially for a non-deficient range, were not causally associated with AF in the general population. Future studies using non-linear design and focusing on the vitamin D deficiency population are needed to further evaluate the causal effect of vitamin D concentrations on AF.

## Introduction

Atrial fibrillation (AF) is the most common persistent cardiac rhythm disorder encountered in clinical practice, and is associated with increased risks of stroke, dementia, heart failure (HF), and death (1). Currently, the estimated prevalence of AF in adults is between 2 and 4% and is strongly associated with advancing age, placing extensive economic and societal burden in terms of morbidity, mortality, increased healthcare resource utilization and loss of productivity (2, 3).

Vitamin D is an essential nutrient obtained from exposure to sunlight, diet, and dietary supplements (4). In the skin, solar ultraviolet B radiation converts 7-dehydrocholesterol to previtamin D_3_, which is immediately isomerized to vitamin D_3_. This precursor form of vitamin D is metabolized to 25-hydroxyvitamin D [25(OH)D] in the liver, which is used to determine vitamin D status in the routine clinical setting. 25-hydroxyvitamin D is biologically inactive and must be converted to its active form 1,25-dihydroxyvitamin D by 25-hydroxyvitamin D-1-α-hydroxylase mainly in the kidneys (4). Vitamin D deficiency is a global problem, as many as 37% of the general population worldwide having vitamin D deficiency under the definition of <20 ng/mL (<50 nmol/L). Using a definition of <12 ng/mL (<30 nmol/L), approximately 7% of the population may be at risk to manifest symptoms due to severe vitamin D deficiency (5).

In some observational studies, a low vitamin D level has been linked with a variety of cardiovascular diseases such as coronary artery disease (CAD), HF and AF (6, 7). However, these associations remain controversial. Moreover, given the nature of observational studies, these results may be confounded by multiple variables. Mendelian randomization (MR) uses genetic variants as instrumental variables to make causal inferences, avoiding potential biases caused from confounding and reverse causation in observational studies. Therefore, a MR study can be considered as analogous to a randomized controlled trial (RCT), inferring a causal association of the risk factor on the disease outcome (8). Whilst current evidence shows that there may be a relationship between vitamin D deficiency and AF, incomparable study designs and methodological limitations make existing evidence less reliable. In this study, we aimed to perform a two-sample MR analysis to determine the potential role of circulating vitamin D status on AF risk.

## Methods

### Study Design and Data Sources

**Figure 1:**
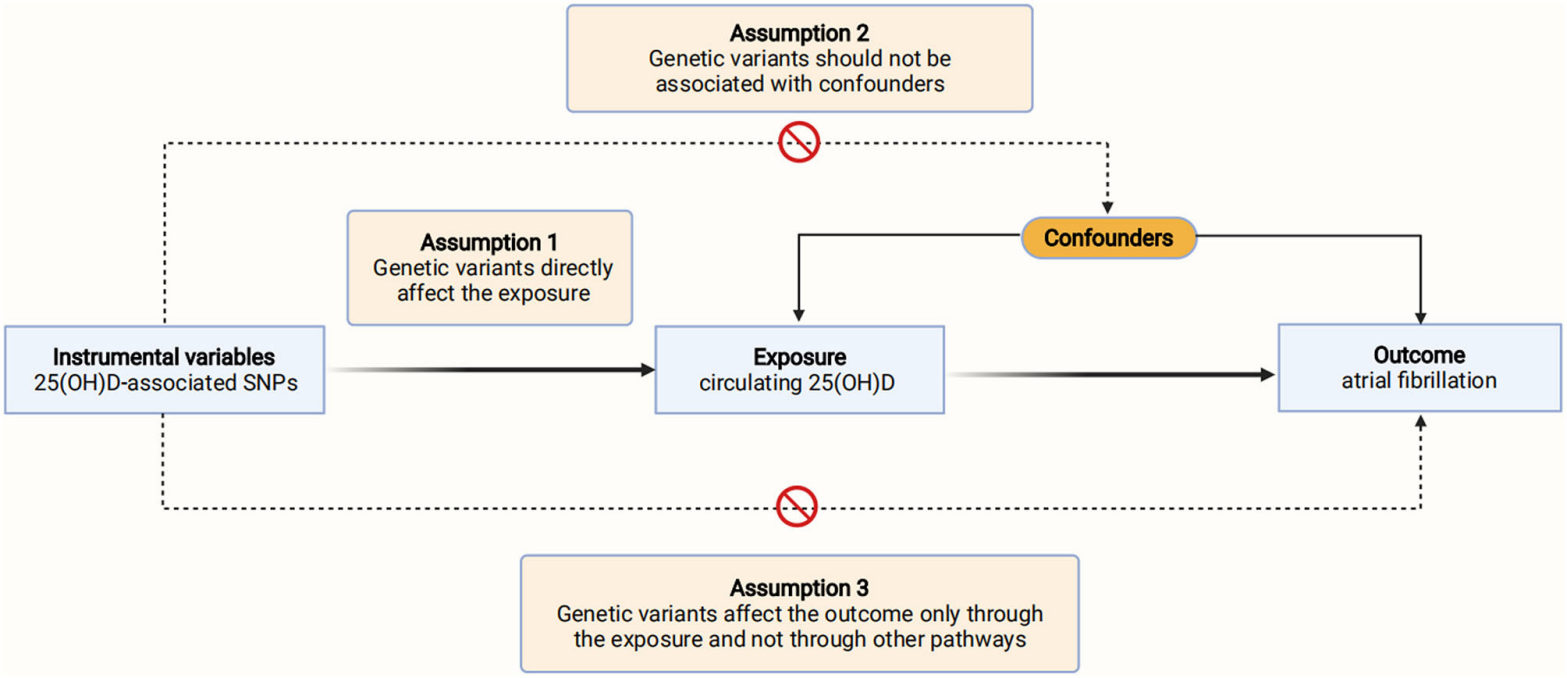
Schematic diagram of the Mendelian randomization assumptions. AF, atrial fibrillation; SNPs, single-nucleotide polymorphisms; 25(OH)D, 25-hydroxyvitamin D.

This Mendelian randomization was reported in accordance with the Strengthening the Reporting of Observational Studies in Epidemiology Using Mendelian Randomization (STROBE-MR) guidelines (9). We performed a two-sample MR analysis to evaluate the causal effect of 25(OH)D concentrations on AF. Three assumptions need to be met to ensure a valid instrumental variable (Figure 1) (8). First, the genetic variants utilized as instrumental variables should be associated with the exposure of interest. Second, the genetic variants should not be associated with confounders. Third, the genetic variants should affect the outcome only through the exposure, but not by other pathways. Data on the association of genetic variants with 25(OH)D and AF were obtained from recently published genome-wide association studies (GWAS) (10, 11). The specific ethical reviews and informed consent had been obtained in the original studies.

### Selection of Genetic Variants

Summary statistics on the association between genetic variants and 25(OH)D concentrations from the recently published SUNLIGHT meta-GWAS were retrieved (10). This study is a large, multicenter, genome-wide association study involving 31 cohorts and case-control studies in Europe, Canada and USA, which was conducted through two stages. The first stage involved a discovery meta-analysis on a total of up to 79,366 European-ancestry individuals and the second stage replicated novel findings in 42,757 independent European-ancestry individuals (10). In the SUNLIGHT meta-GWAS, additive genetic models using linear regression on natural-log transformed 25(OH)D were fitted and a fixed-effect inverse variance weighted meta-analysis was performed across the contributing studies. Detailed descriptions of these 31 participating studies are presented in Supplementary Table 1, and the mean serum vitamin D concentrations of these studies were mostly in a non-deficient range. Information regarding the eligibility criteria, quality control, power calculation, genotyping platform and 25(OH)D measurements methods applied in each included study have been reported previously (10). Six SNPs were identified from the SUNLIGHT meta-GWAS at a genome-wide significance level (*P* < 5.0 × 10^−8^) (10). Linkage disequilibrium was not detected after preforming the linkage disequilibrium clumping process (*r*^2^ <0.001, distance >10,000 kb).

Utilizing the GWAS data, the pleiotropic associations between each of the six SNPs with several potential confounders were evaluated, including type 2 diabetes mellitus (T2DM) in 110,452 individuals (Diabetics Genetics Replication and Meta-analysis, DIAGRAM) (12), body mass index (BMI) in 339,224 individuals (Genetic Investigation of Anthropometric Traits, GIANT) (13), systolic and diastolic blood pressure in over 1 million individuals (International Consortium for Blood Pressure, ICBP) (14), alcohol consumption and smoking in up to 1.2 million individuals (GWAS and Sequencing Consortium of Alcohol and Nicotine use) (15). The Phenoscanner tool (http://www.phenoscanner.medschl.cam.ac.uk) was used to evaluate whether these SNPs were associated with other traits (16). The results showed that, at a genome-wide significance level (*P* < 5.0 × 10^−8^), none of these 6 SNPs had pleiotropic associations with T2DM, BMI, blood pressure, alcohol and tobacco use (Supplementary Table 2), or other potential confounders. Thus, six SNPs associated with circulating 25(OH)D concentrations were used as instrumental variables. An F statistic >10 indicated a low risk of weak instrument bias according to the formula: *F* = *R*^2^ × (N-2)/(1-*R*^2^), where *R*^2^ is the proportion of variance in vitamin D instruments using the formula: *R*^2^ = 2 × effect allele frequency × (1 – effect allele frequency) × (Beta/standard deviation (SD, equals 1))^2^ and *N* represents the sample size (17).

### Outcome Data Source

Summary statistics for the associations of the six 25(OH)D-related SNPs with AF were extracted from the GWAS conducted by Nielsen et al. (11) which is currently the largest meta-GWAS focusing on the genetic architecture of AF, comparing a total of 60,620 cases and 970,216 controls of European ancestry from six contributing studies. Detailed descriptions of these 6 participating studies are presented in Supplementary Table 1. Cases were selected using *International Classification of Diseases* codes (ICD-9 or ICD-10), including AF and atrial flutter (11). Information regarding the eligibility criteria, power calculation, genotyping platform and quality control have been reported previously (11).

### Statistical Analysis

Estimation of the overall causal effects between circulating 25(OH)D concentrations and AF was performed using both the fixed-effect and random-effects inverse variance weighted (IVW) method, which assume that all SNPs are valid instrumental variables based on the MR assumptions (18). In order to account for potential violations of the assumptions underlying the IVW method, we performed sensitivity analyses, including the weighted median, MR-Egger, simple median and penalized weighted median methods. The weighted median method provides a consistent effect estimate even when up to 50% of the genetic variants are invalid instruments using the inverse of the variance of the ratio estimates as weights (19). The MR-Egger approach provides a valid effect estimate even if all SNPs are invalid instruments and the intercept could be used to detect directional pleiotropy (20). A zero intercept for MR-Egger (*P* > 0.05) was considered to indicate no pleiotropic bias. The median-based estimator and MR-Egger can obtain consistent causal estimates under weaker assumptions. Whereas, these methods can be sensitive to genetic variants with heterogeneous causal estimates, therefore, the penalized weighted median method was also performed to provide a robust estimate by penalizing the weights of SNPs with heterogeneous causal estimates (21). Mendelian randomization pleiotropy residual sum and outlier (MR-PRESSO) test was performed to detect and correct for horizontal pleiotropic outliers (22). The Cochran Q test for heterogeneity was applied to test the presence of horizontal pleiotropy. The single SNP analysis using the Wald ratio method and leave-one-out sensitivity analysis were conducted to determine whether the association between 25(OH)D and AF was affected by single SNP. The odds ratios (ORs) are scaled per unit change in natural-log-transformed 25(OH)D level.

Power calculations were conducted using the mRnd power calculation tool (23). Given a sample size of 1,030,836 and 2.84% of variance in 25(OH)D explained by the genetic variants, this study had 80% power at an alpha rate of 5% to detect an OR of 0.931 for AF per unit increase in log 25(OH)D.

A two-side *P* < 0.05 was considered statistically significant in statistical tests for MR analyses. All statistical analyses were conducted using the “TwoSampleMR” package (24) and “MRPRESSO” package in the R software (version 4.0.4, R Development Core Team, Vienna, Austria).

## Results

The characteristics of 6 SNPs and their association with 25(OH)D and AF are presented in Table 1. None of the individual 6 SNPs was associated with AF at Bonferroni corrected significance level *P* < 0.008 [(*P* < 0.05)/6 SNPs], and the allele frequencies between the exposure and outcome population were generally similar (Table 1). The F-statistic of each SNPs was more than 10, which indicated that they were all strong instrumental variables. There was no sample overlap between the exposure and outcome population (Table 1).

**Table 1:** Characteristics of the 25(OH)D-associated SNPs.

**SNP**	**Position**	**Nearby gene**	**EA/NEA**	**25(OH)D**					**Atrial fibrillation**		
				**EAF**	***R*** **^2^ (%)**	***F*** **-statistic**	**Beta (SE)**	***P*** **-value**	**EAF**	**Beta (SE)**	***P*** **-value**
rs3755967	4:72828262	GC	T/C	0.28	0.319	254.28	−0.089 (0.0023)	4.74 × 10^−343^	0.28	0.0056 (0.0074)	0.45
rs12785878	11:70845097	NADSYN1/ DHCR7	T/G	0.75	0.049	38.59	0.036 (0.0022)	3.80 × 10^−62^	0.70	0.0011 (0.0075)	0.89
rs10741657	11:14871454	CYP2R1	A/G	0.40	0.046	36.63	0.031 (0.0022)	2.05 × 10^−46^	0.58	−0.0117 (0.0067)	0.08
rs17216707	20:52165769	CYP24A1	T/C	0.79	0.022	17.81	0.026 (0.0027)	8.14 × 10^−23^	0.19	−0.0156 (0.0087)	0.07
rs10745742	12:94882660	AMDHD1	T/C	0.39	0.017	20.98	0.019 (0.0020)	2.10 × 10^−20^	0.38	−0.0074 (0.0068)	0.28
rs8018720	14:38625936	SEC23A	C/G	0.82	0.011	13.02	−0.019 (0.0027)	1.11 × 10^−11^	0.81	0.0058 (0.0088)	0.51

*The beta coefficients for the association between SNPs and 25(OH)D are based on per effect allele per unit change in log 25(OH)D*.

*EA, effect allele; EAF, effect allele frequency; NEA, non-effect allele; SE, standard error; SNP, single nucleotide polymorphism*.

**Figure 2:**
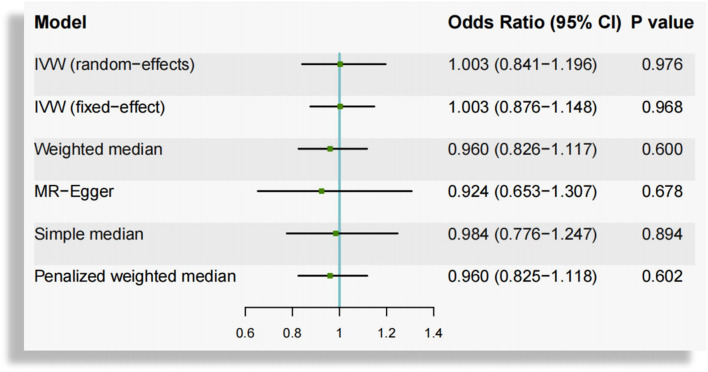
Mendelian randomization estimates of the association between genetically predicted circulating vitamin D and atrial fibrillation. CI, confidence interval; IVW, inverse-variance-weighted.

In the overall analysis based on IVW method, the OR of AF per unit increase in log 25(OH)D was 1.003 (95% CI, 0.841–1.196; *P* = 0.976) for random-effects method and 1.003 (95% CI, 0.876–1.148; *P* = 0.968) for fixed-effect method, which was consistent with the results from weighted median [OR, 0.960, 95% CI (0.826–1.117); *P* = 0.600], MR-Egger [OR, 0.924, 95% CI (0.653–1.307); *P* = 0.678], simple median [OR, 0.984, 95% CI (0.776–1.247); *P* = 0.894] and penalized weighted median method [OR, 0.960, 95% CI (0.825–1.118); *P* = 0.602], indicating that the circulating 25(OH)D concentrations were not significantly associated with AF (Figure 2). Scatterplot depicting the relationship of the SNP effects on the 25(OH)D against the SNP effects on AF is shown in Supplementary Figure 1.

**Figure 3:**
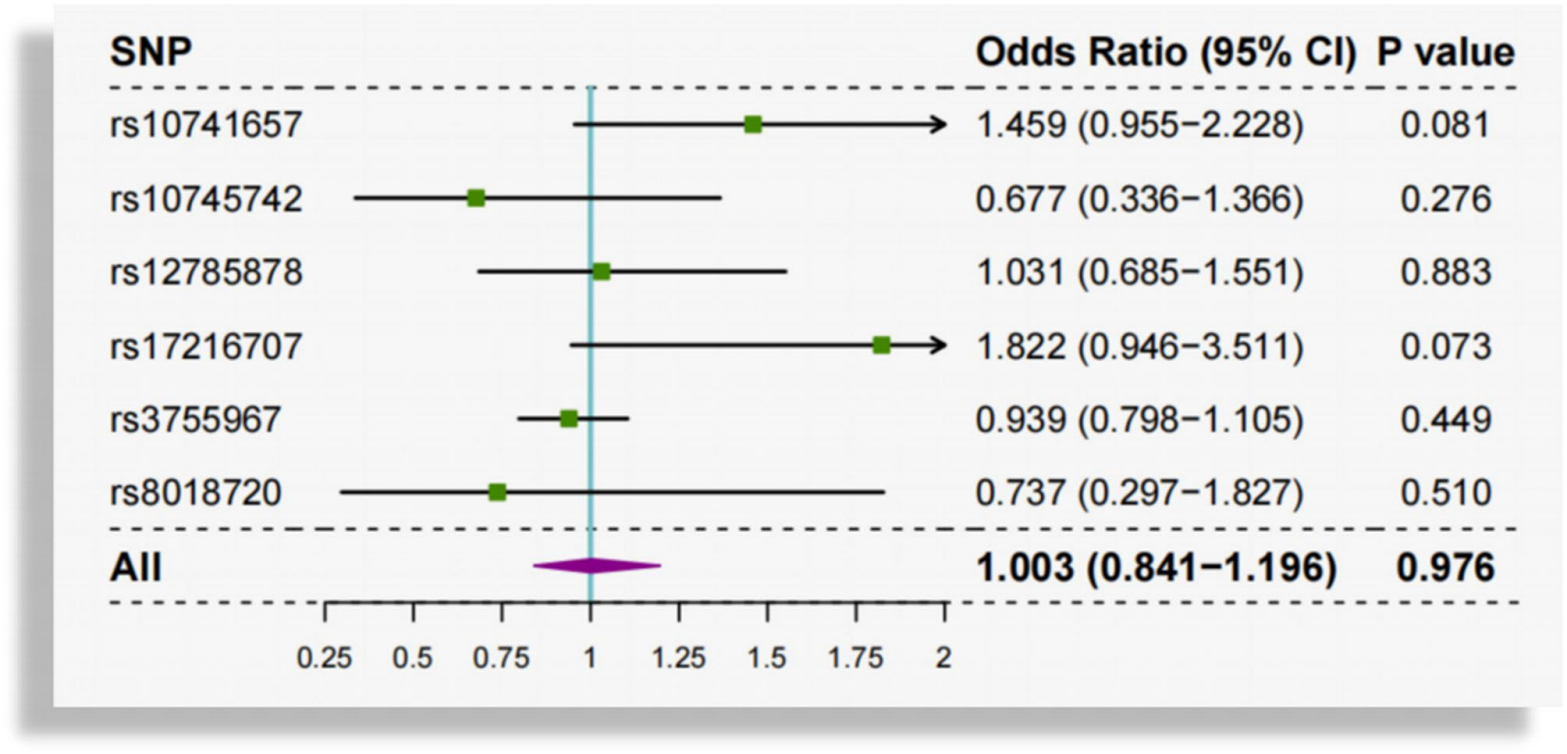
Single SNP analysis of genetically predicted circulating vitamin D and atrial fibrillation. SNP, single-nucleotide polymorphism.

Heterogeneity among the causal estimates of the six SNPs was not observed (*Q* = 8.476, *P* = 0.132). There was no evidence of pleiotropy using MR-Egger (intercept = 0.004, se = 0.008, *P* = 0.610) or MR-PRESSO global test (*P* = 0.312). No outlier SNPs were identified in the MR-PRESSO analysis. According to leave-one-out analysis (Supplementary Figure 2) and single SNP analysis (Figure 3), the association between circulating 25(OH)D and AF was not driven by any individual SNP.

## Discussion

In the present MR analysis, we have capitalized on the summary statistics of two large meta-GWAS conducted for serum 25(OH)D and AF in European populations, without any sample overlap, and constructed a strong instrumental variable for 25(OH)D from six SNPs. We have employed a range of MR methods to investigate the association between 25(OH)D and AF. However, none of these analyses suggested a causal relationship between circulating 25(OH)D concentrations and AF in the general population.

As an essential nutrient for individuals, vitamin D performs an important role in the regulation of calcium, phosphorus, and bone metabolism. Besides, vitamin D receptors (VDR) are found in a variety of cells and tissues, including endothelial cells, vascular smooth muscle cells, cardiac myocytes and fibroblasts (25). Thus, it exerts many other important cellular regulatory functions include modulation of oxidative stress status, inflammatory response, mitochondrial function, insulin, and renin-angiotensin–aldosterone system (RAAS) (25).

In experimental study, Hanafy et al. (26) found that vitamin D increased the action potential duration and contractility in isolated rabbit left atrium tissue, revealed a direct electromechanical effect of vitamin D on preventing or terminating AF. Moreover, in our previous meta-analysis investigating the relationship between circulating vitamin D levels and AF risk which included observational studies mainly assessing for chronic AF, the results suggested that vitamin D deficiency modestly increases the risk of AF (7). More recently, another meta-analysis of observational studies also suggested that serum vitamin D deficiency (<20 ng/ml) was associated with an increased risks of AF in the general population and postoperative AF in patients underwent coronary artery bypass graft (CABG) (27). However, in the VITAL study (28), a large randomized clinical trial that recruited 25,119 adults for a median 5.3 years of daily doses (2,000 IU) of vitamin D supplementation and follow-up, the results did not support the use of vitamin D for the primary prevention of new-onset AF. Therefore, data concerning the causal association between vitamin D and AF are so far contradictory and controversial.

Currently, there are various studies looking at cardiovascular effects of vitamin D using MR, designed to minimize bias from confounding, however, most of these studies have found null effects (29). For example, a one-sample MR in 92,416 individuals of Danish descent failed to demonstrate any evidence of a causal association between plasma 25(OH)D levels and CAD or myocardial infarction (30). Similarly, a 2020 study in 417,580 Europeans from the UK Biobank found no evidence to suggest that vitamin D concentration is associated with CAD (31). In regarding to hypertension, the evidence from existing MR studies on the effects of predicted serum 25OHD levels on hypertension, systolic and diastolic blood pressure, consistently does not support any of these outcomes (29). In the current study, we also found no evidence that genetically predicted 25(OH)D is associated with increased risk of AF in the general population. Results of these MR studies suggests that the observed association of reduced 25(OH)D with CAD and AF may not represent a causal relationship, but more likely is due to confounding by unaccounted influences or due to reverse causation.

Notably, a recent non-linear MR study have suggested an L-shaped association of 25(OH)D with CVD risk, with increased CVD risk largely restricted to individuals with low vitamin D status (32). Moreover, in another similar study (33), the authors have demonstrated non-linear dose-response relationships of 25(OH)D with CAD, stroke and all-cause mortality in observational analysis, and identified an association of genetically-predicted 25(OH)D with all-cause mortality only for individuals with vitamin D deficiency (<25 ng/mL) but not for general population. Therefore, further MR studies using non-linear design and focusing on the vitamin D deficiency population are needed. Besides, categorical analysis using different cutpoints proposed for vitamin D deficiency is also warranted in order to identify the subsets of individuals who may benefit more from vitamin D supplementation. Additionally, in a MR study conducted in a small sized Chinese cohort of cardiac outpatients with stable CAD, in contrast to our study that evaluated individuals primarily form European ancestry, the result suggested genetically deprived vitamin D exposure is associated with increased risk of AF (34). Thus, the null effects observed in our study may also be due to the racial disparity. Future studies focusing the effects of vitamin D on risk of AF among different ethnic groups are needed.

### Strengths and Limitations

This study has several strengths. First, using Mendelian randomization method, this study could provide a robust estimate of causal relationships between vitamin D and AF. Second, there was no sample overlap between studies included in the GWAS meta-analysis of vitamin D and AF, which could help reduce the severity of weak instrument bias. Third, the individuals included in these two GWAS meta-analysis were primarily of European ancestry, and the effect allele frequencies of the 6 vitamin D-associated SNPs were very similar between the two populations, which could mitigate the potential effect of population stratification. The results of this study also should be interpreted in conjunction with some limitations. First, though several approaches were conducted to assess and adjust for potential confounding or pleiotropic effects, we could not completely rule out the influence of unknown potential confounders. Second, access to only summarized data limits the range of analyses that can be performed, such as subgroup analyses, non-linear analysis and categorical analysis using different cutpoints for vitamin D deficiency.

## Conclusion

Genetically predicted circulating vitamin D concentrations, especially for a non-deficient range, were not causally associated with AF in the general population. Future studies using non-linear design and focusing on the vitamin D deficiency population are needed to further evaluate the causal effect of 25(OH)D concentrations on AF.

## Data Availability Statement

The original contributions presented in the study are included in the article/Supplementary Material, further inquiries can be directed to the corresponding authors.

## Ethics Statement

The ethical reviews and informed consent had been obtained in the original studies, and were not required for the current analysis.

## Author Contributions

All authors listed have made a substantial, direct, and intellectual contribution to the work and approved it for publication.

## Funding

This work was supported by grants from the National Natural Science Foundation of China (Grant Number: 82100341 to ZZ, 81970270 and 82170327 to TL).

## Conflict of Interest

GYHL has been a consultant and speaker for BMS/Pfizer, Boehringer Ingelheim and Daiichi-Sankyo. No fees are received personally. The remaining authors declare that the research was conducted in the absence of any commercial or financial relationships that could be construed as a potential conflict of interest.

## Publisher's Note

All claims expressed in this article are solely those of the authors and do not necessarily represent those of their affiliated organizations, or those of the publisher, the editors and the reviewers. Any product that may be evaluated in this article, or claim that may be made by its manufacturer, is not guaranteed or endorsed by the publisher.
