# Supplementary material for: Circulating Vitamin D Concentrations and Risk of Atrial Fibrillation: A Mendelian Randomization Study Using Non-deficient Range Summary Statistics
Source: Front Nutr. 2022 Jun 17;9:842392. doi: 10.3389/fnut.2022.842392 (PMC9248862; doi:10.3389/fnut.2022.842392)
Supplement: Supplementary file 1 [file Data_Sheet_1.docx]

**Supplementary Material**

**
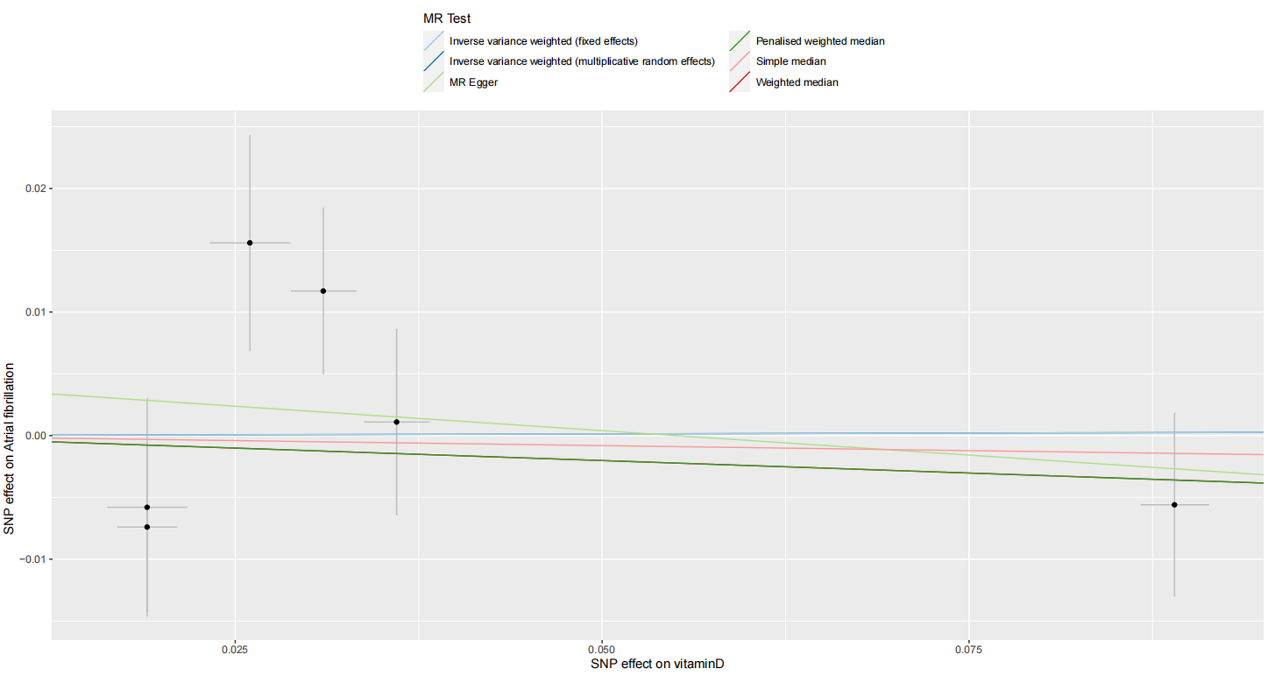
**

**Supplementary Figure 1**. Scatterplot of the effect size of each SNP on circulating vitamin D and atrial fibrillation. Abbreviation: SNP, single-nucleotide polymorphism.

**
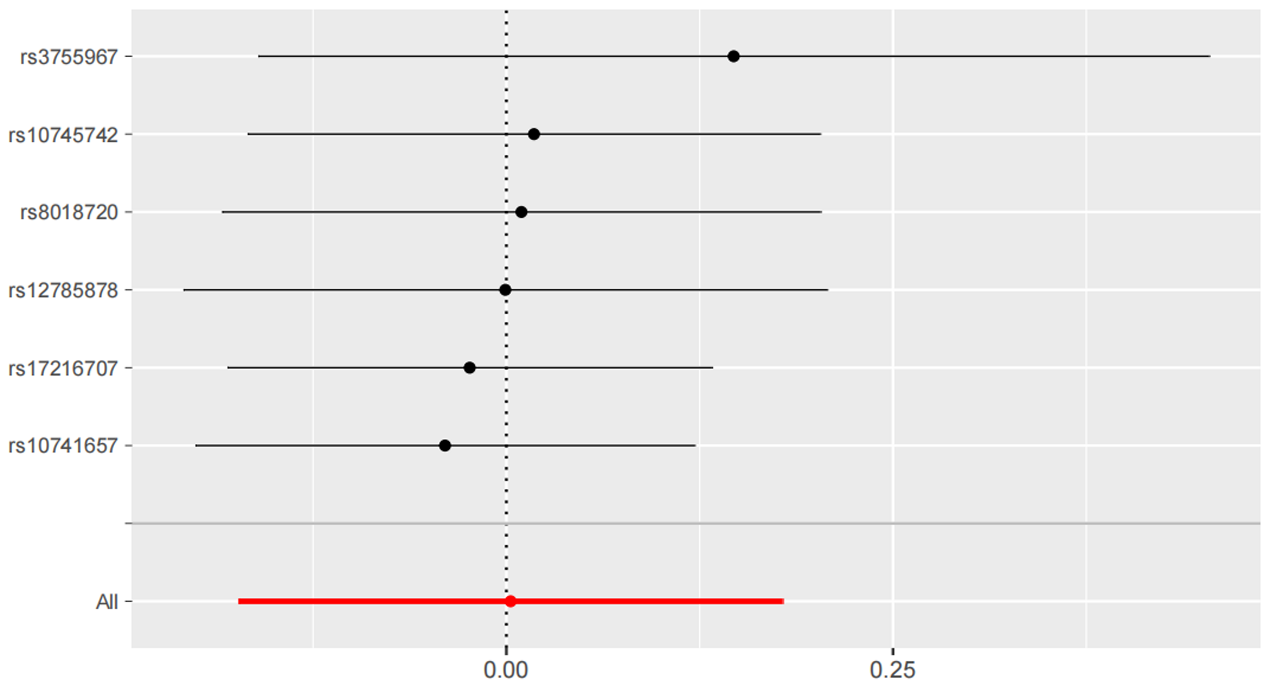
**

**Supplementary Figure 2.** Leave-one-out analysis of genetically predicted circulating vitamin D and atrial fibrillation.

**Supplementary Table 1 Characteristics of the individual studies included in the meta-GWAS**

| **Studies** | **Design** | **Sample size** | **Genotyping platform** | **Vitamin D measurements or diagnosis of AF** | **Mean**  **vitamin D**  **(sd), nmol/L** |
| --- | --- | --- | --- | --- | --- |
| **GWAS for vitamin D** | | | | | |
| 1958 British Birth Cohort (1958BC) | Population cohort | 4985 | Affymetrix 500K or Illumina 550K | ELISA | 58 (25.2) |
| Cardiovascular Health Study (CHS) | Population cohort | 1791 | Illumina 317K CNV chip | high-performance liquid chromatography-tandem mass spectrum | 66 (25.6) |
| Framingham Heart Study (FHS) | Population cohort | 5654 | Affymetrix 500K and MIPS 50K | radioimmunoassay | 78 (37.3) |
| Gothenburg Osteoporosis and Obesity Determinants Study (GOOD) | Population cohort | 921 | Human610-Quadv1 | radioimmunoassay | 65 (22.0) |
| Health, Aging, and Body Composition Study (Health ABC) | Population cohort | 1558 | Illumina 1M | radioimmunoassay | 72 (27.6) |
| The Study of Indiana Women (Indiana) | Population cohort | 567 | Human610-Quadv1 | high-performance liquid chromatography-tandem mass spectrum | 73 (32.7) |
| North Finland Birth Cohort 1966 (NFBC) | Population cohort | 4604 | Illumina Infinium cnvDuo array | high-performance liquid chromatography-tandem mass spectrum | 62 (23.9) |
| Old Order Amish Study (OOA) | Population cohort | 330 | Affymetrix 500K and 100K | radioimmunoassay | 57 (18.7) |
| The Rotterdam Study (RS) | Population cohort | 9550 | HumanHap550K | radioimmunoassay | 66 (27.7) |
| TwinsUK | Population cohort | 5135 | Illumina 317K or 610K | radioimmunoassay | 71 (32.9) |
| Alpha-Tocopherol, Beta- Carotene Cancer Prevention Study (ATBC) | Case-control study | 1372 | Illumina 550K (or higher version) platform | radioimmunoassay and chemiluminescence immunoassay | NA |
| Atherosclerosis Risk In Communities (ARIC) | Population cohort | 8124 | Affymetrix 6.0 array | liquid chromatography tandem high-sensitivity mass spectrometry | NA |
| AtheroGene registry | Population cohort | 1062 | Illumina array | radioimmunoassay | NA |
| B-vitamins for the Prevention Of Osteoporotic Fractures (B-PROOF) | Population cohort | 2525 | IlluminaOmniExpression array | liquid chromatography-tandem mass spectrometry | NA |
| Epidemiology of Diabetes  Interventions and Complications (EDIC) | Population cohort | 1094 | Human 1M BeadChip | Liquid chromatography-tandem mass spectrometry | NA |
| Case-Control Study for Metabolic Syndrome (GenMets) | Case-control study | 1641 | Illumina 610K | radioimmunoassay | NA |
| Helsinki Birth Cohort Study (HBCS) | Population cohort | 917 | Illumina 670K | high-performance liquid chromatography | NA |
| Coronary Heart Disease Case Control Study nested within NHS and HPFS (HPFS_CHD) | Case-control study | 1245 | Illumina 550K (or higher version) platform | radioimmunoassay | NA |
| Breast Cancer Case-Control Study nested within NHS (NHS_BRCA) | Case-control study | 870 | Illumina 550K (or higher version) platform | radioimmunoassay | NA |
| Invecchiare in Chianti Study (InChianti) | Population cohort | 1094 | Illumina Infinium HumanHap 550 chip | Enzyme immunoassay (OCTEIA 25-hydroxyvitamin D kit) | NA |
| Cooperative Health Research in the region Augsburg (KORA) | Population cohort | 1805 | Affymetrix 6.0 array | Enhanced chemiluminescence immunoassay | NA |
| Leiden Longevity Study (LLS) | Population cohort | 2265 | Illumina Infinium HD Human660W-Quad and Illumina OmniExpress BeadChips | Electrochemiluminescence immunoassays | NA |
| Ludwigshafen Risk and Cardiovascular Health Study (LURIC) | Population cohort | 2846 | Affymetrix 6.0 array | radioimmunoassay | NA |
| Multi-Ethnic Study of Atherosclerosis (MESA) | Population cohort | 2240 | Affymetrix 6.0 array | high-performance HPLC-tandem mass spectrometry | NA |
| Nijmegen Biomedische Studie (NBS) | Population cohort | 2610 | Illumina HumanHap300 | radioimmunoassay | NA |
| Orkney Complex Disease Study (ORCADES) | Population cohort | 847 | Illumina HumanHap300 | Liquid chromatography tandem mass spectrometry | NA |
| Prostate, Lung, Colorectal, and Ovarian Cancer Screening Trial (PLCO) | Case-control study | 1315 | Illumina 240K, 300K, and 550K platform | radioimmunoassay | NA |
| PROspective Study of Pravastatin in the Elderly at Risk (PROSPER) | Population cohort | 4871 | Illumina 660-Quad BeadChip | Electrochemiluminescence immunoassays | NA |
| Study of Health in Pomerania (SHIP) | Population cohort | 1655 | Affymetrix 500K array | IDS-iSYS 25-Hydroxy Vitamin D assay | NA |
| Scottish Colorectal Cancer Study (SOCCS) | Case-control study | 1165 | Custom Illumina Infinium array | Liquid chromatography tandem mass spectrometry | NA |
| Cardiovascular Risk in Young Finns Study (YFS) | Population cohort | 1984 | Illumina Human 670K BeadChip | radioimmunoassay | NA |
| **GWAS for atrial fibrillation** | | | | | |
| The Nord-Trøndelag Health Study (HUNT) | Population cohort | 69635 | Illumina HumanCore Exome v1.0 and v1.1 | ICD-10 code I48 or ICD-9 code 427.3 | / |
| deCODE | Population cohort | 371632 | Illumina chips | ICD-10 code I48 or ICD-9 code 427.3 | / |
| Michigan Genomics Initiative (MGI) | Population cohort | 12275 | Illumina Human Core Exome v1.0 and v1.1 | ICD-9 code 427.31 | / |
| DiscovEHR | Population cohort | 48482 | Illumina Human OmniExpress Exome Beadchip | At least 1 electronic health record problem list entry or at least 2 diagnosis code entries for 2 separate clinical encounters on separate calendar days for ICD-10 I48 | / |
| UK Biobank | Population cohort | 395739 | Applied Biosystems UK BiLEVE Axiom Array and UK BioBank Axiom Array | ICD-9 427.3 or ICD-10 I48 | / |
| AFGen Consortium | Meta-GWAS study | 133073 | Illumina Human Hap550 v3 or Hap610 v1 | ICD-9, ICD-10, or 12-lead ECG at the examinations | / |

AF, atrial fibrillation; ECG, electrocardiogram; ELISA, enzyme-linked immunosorbent assay; GWAS, genome-wide association studies; ICD, International Classification of Diseases; NA, not available.

**Supplementary Table 2 P values for the association of 25(OH)D-associated SNPs with potential confounders**

| SNP | Gene | BMI | T2DM | SBP | DBP | Alcohol consumption | Smoking |
| --- | --- | --- | --- | --- | --- | --- | --- |
| rs3755967 | GC | 0.51 | 0.05 | 0.60 | 0.51 | 0.47 | 0.82 |
| rs12785878 | NADSYN1/DHCR7 | 0.69 | 0.21 | 0.54 | 0.06 | 0.42 | 0.48 |
| rs10741657 | CYP2R1 | 0.02 | 0.67 | 2.64×10^-7^ | 0.0001 | 0.13 | 0.02 |
| rs17216707 | CYP24A1 | 0.28 | 0.01 | 0.15 | 0.01 | 0.10 | 0.27 |
| rs10745742 | AMDHD1 | 0.83 | 0.15 | 0.04 | 0.46 | 0.49 | 0.98 |
| rs8018720 | SEC23A | 1.00 | 0.17 | 0.41 | 0.29 | 0.99 | 0.22 |

BMI, body mass index; DBP, diastolic blood pressure; SBP, systolic blood pressure; SNP, single-nucleotide polymorphism; T2DM, type 2 diabetes mellitus.
